# Does acupuncture work? Is there any evidence?

**DOI:** 10.1590/S1516-31802006000300001

**Published:** 2006-05-04

There is no lack of evidence regarding acupuncture. A search in PubMed just for clinical trials brings up 3,707 references on acupuncture. If the search is for systematic reviews over the last five years alone, around 250 articles will be identified.

As is well known, not all the 3,707 articles located as clinical trials will in fact be this type, and the same goes for systematic reviews. There will be a need to critically evaluate each reference and separate the wheat from the chaff.

It would thus be better to search from the starting point of a question. The first step should be to formulate specific questions. Each question will require at least the following items:

1. The health problem
2. The intervention (type of acupuncture)
3. Control intervention (placebo, false acupuncture, waiting list, drugs, etc.)
4. The outcome (cure, disappearance of the pain, return to work, etc.).

Another strategy that is very efficient is to put these words that form the question in the search location for the Cochrane Library, which has been free to access in Brazil for around five years (www.bireme.br/cochrane). Doctors and lay people can access it, and there is an article in Diagnóstico e Tratamento^1^ that gives guidance on how to better exploit the Cochrane Library.

In the Cochrane Library, readers will find 13 completed systematic reviews (best level of evidence) and 12 clinical protocols, i.e. systematic reviews that are in progress, for which the complete text is expected to become available within six to twelve months, approximately, about acupunture.

Only two of the completed reviews in the Cochrane Library^2–13^ present results that provide support for the use of acupuncture: acupuncture for post-chemotherapy nausea and vomiting without the use of antihemetic drugs, and for idiopathic headache. Other reviews show a lack of convincing effects from acupuncture in relation to smoking, cocaine dependence, schizophrenia treatment, etc.

In many cases, there is a lack of evidence to base decisions on. Lack of evidence must not, however, be confused with lack of effects. Lack of evidence means that the therapy has not been studied through adequate clinical trials. Lack of effect means that randomized controlled studies with adequate sample sizes, adequate control groups, reliable measurements of outcomes, etc., have been conducted but that improvements were not observed more frequently in the treated group than in the control group.

For professionals who are looking for good evidence in order to know whether or not to refer a patient for acupuncture treatment, this may be bad news. This is also the situation with a good proportion of allopathic treatments, many of which extremely expensive and barbaric.

On the other hand, professionals who wish to contribute towards medical knowledge and towards enlightening health-care through new knowledge may conduct controlled clinical trials to evaluate acupuncture or allopathic therapy, since these open the door, if not to paradise then to an extremely important and fruitful professional activity.

So, what purpose have the completed systematic reviews served in relation to answering specific questions? After all, these reviews have not found enough clinical trials to constitute evidence in favor of or against such procedures. Nonetheless, they have served exactly this purpose: to show that more and better clinical trials are needed on this subject. And they have shown what characteristics such studies must have in order to definitively reduce the uncertainties to a point at which there will be reasonable evidence regarding the effectiveness, efficiency and safety of each treatment or preventive measures. In this respect, the Cochrane Collaboration has contributed greatly, through establishing the basis for true scientific progress for the benefit of human health.

## HOMAGE

One final note: On June 1, 2006, an exhibition began in the National Portrait Gallery in London, prepared by the photographer Julia Fullerton-Batten, in homage to the 15 scientists who contributed most towards improving people's lives over the past century. The exhibition continues in London until October. The Founder of the Cochrane Collaboration, Sir Iain Chalmers, was one of those chosen. He refused to accept this homage alone and demanded that those who had collaborated through their work should also be represented with their photographs. The present humble collaborator thanks him for the honor of being remembered as part of the group receiving the homage, but also acknowledges that everyone who has collaborated with us in Brazil and abroad also deserves similar homage. Since the Brazilian Cochrane Center reaches its tenth anniversary this year, we will attempt to do the same, by paying homage to all friends and collaborators.
